# Multiomics comparative analysis of the maize large grain mutant *tc19* identified pathways related to kernel development

**DOI:** 10.1186/s12864-023-09567-z

**Published:** 2023-09-11

**Authors:** Qing Cai, Fuchao Jiao, Qianqian Wang, Enying Zhang, Xiyun Song, Yuhe Pei, Jun Li, Meiai Zhao, Xinmei Guo

**Affiliations:** 1 College of Agronomy, Qingdao Agricultural University, Qingdao, 266109 China; 2 The Characteristic Laboratory of Crop Germplasm Innovation and Application, Provincial Department of Education, College of Agronomy, Qingdao Agricultural University, Qingdao, 266109 China; 3 College of Life Sciences, Qingdao Agricultural University, Qingdao, 266109 China

**Keywords:** Maize, Large gain mutant, Multiomics, Comparative analysis, Pathways

## Abstract

**Background:**

The mechanism of grain development in elite maize breeding lines has not been fully elucidated. Grain length, grain width and grain weight are key components of maize grain yield. Previously, using the Chinese elite maize breeding line Chang7-2 and its large grain mutant *tc19*, we characterized the grain size developmental difference between Chang7-2 and *tc19* and performed transcriptomic analysis.

**Results:**

In this paper, using Chang7-2 and *tc19*, we performed comparative transcriptomic, proteomic and metabolomic analyses at different grain development stages. Through proteomics analyses, we found 2884, 505 and 126 differentially expressed proteins (DEPs) at 14, 21 and 28 days after pollination, respectively. Through metabolomics analysis, we identified 51, 32 and 36 differentially accumulated metabolites (DAMs) at 14, 21 and 28 days after pollination, respectively. Through multiomics comparative analysis, we showed that the phenylpropanoid pathways are influenced at transcriptomic, proteomic and metabolomic levels in all the three grain developmental stages.

**Conclusion:**

We identified several genes in phenylpropanoid biosynthesis, which may be related to the large grain phenotype of *tc19*. In summary, our results provided new insights into maize grain development.

**Supplementary Information:**

The online version contains supplementary material available at 10.1186/s12864-023-09567-z.

## Introduction

Maize (*Zea mays* L.) is an important food, feed, and fuel crop worldwide. Improving grain yield is a top priority in modern breeding [1]. Grain yield is determined by grain size, composed of three secondary traits: grain length, gain width and grain thickness [2]. The mature kernels of maize consist of endosperm and embryos, where storage of abundant starch and protein [3].

Transcriptomic analysis of nucellus (embryo sac included) collected at an interval of 4 or 6 h within the first six days of seed development, a total of 22,790 expressed genes were identified, enriched in calcium signaling, nucleosome, auxin response and mitosis pathways [4]. Using the dynamic transcriptomic data of 53 maize samples from the beginning of pollination to 38 days after pollination, more than 20,000 seed expressed genes were found, and zein and starch synthesis genes are the major contributors to endosperm expressed transcripts [5]. Additionally, comparative proteomics and metabolic analysis have been used to identify new pathways affecting grain development in maize [6]. These indicate that by using transcriptomic data and combing multiomics analysis, we can reveal biological mechanisms systematically.

Defective or empty grain mutants hve been identified to clone genes responsible for maize grain development. For example, abnormal expression of genes encoding pentatricopeptide repeat (PPR) proteins, which have key functions in the mitochondrial electron transport chain, are the main factors leading to defective grains [7–11]. Other processes, such as RNA transcription and processing also involved the grain development regulation [12–15]. However, gene clone using large grain mutants have been rarely reported.

Many grain growth-related quantitative trait loci (QTLs) have been identified in crops [16]. Several homologs of rice genes related to grain development are functionally confirmed in maize. For example, *GS3* is a major QTL gene for grain length and weight in rice [17]. Its maize homolog *ZmGS3* also controls maize grain weight [18]. *GS5* is an important gene for rice grain width development [19], and *ZmGS5* also affects maize grain development [20]. Recently, a few QTL genes for kernel-related traits have been identified via map-based cloning in maize. Exampled as the retromer protein ZmVPS29 regulates maize kernel morphology likely through auxin-dependent processes [21].

The mechenaim of grain development is not fully understood, one of the reasons is the limitation of the maize materials being studied. Mutants with a large grain phenotype have been rarely identified and characterized. We previously identifed a large grain mutant *tc19* on the background of the Chinese maize elite breeding line Chang7-2. We observed that *tc19* shows different grain size and grain growth rates with Chang7-2 and identified several genes related to hormone signal pathways using transcriptomic analysis [22]. Here, by combining transcriptomic, proteomic and metabolomic analysis, we aim to identify new pathways and provide insights for maize grain development in *tc19*.

## Materials and methods

### Plant materials and phenotyping

Plant materials and phenotyping were performed same as previously [22]. Maize inbred line Chang7-2 and its large grain mutant *tc19* were selected from the Maize Molecular Breeding Laboratory of Qingdao Agricultural University. Fifteen rows of Chang7-2 and *tc19* were planted in the Jiaozhou experimental station of Qingdao Agricultural University on May 2018. The row length is 3 m, the row spacing is 0.6 m, and the plant spacing is 0.2 m. Eight rows of Chang7-2 and *tc19* were planted in the Pingdu experimental station of Qingdao Agricultural University on April 2019. The row length is 9 m, the row spacing is 0.6 m, and the plant spacing is 0.2 m. The maize plants were pollinated manually. For grain phenotyping, at least 10 ears of each line are selected at 14, 21, 28 DAP and the mature stage. At least three biological replicates were performed. Statical analysis was performed by using Excel 2010 and DPS 17.10.

### Proteomic analysis

The samples used for phenotyping in Pingdu experimental station were also collected for proteomic analysis. Ears were taken at 7 days, 14 days, 21 days and 28 days after pollination. Three biological replicates were used for each stage. Grains were isolated from the center of the ears and immediately frozen in liquid nitrogen. DIA proteomics test and data analysis were performed by GENE DENOVO in Guangzhou, China. Total proteins were extracted using the cold acetone method [23]. Protein quality was examined with SDS-PAGE. The concentration of protein in the supernatant was measured by using the BCA Protein Assay Kit. 50 µg proteins were suspended in 50 µl solution, add 1 µl 1 M dithiothreitol, incubated at 55 °C for 1 h, add 5 µl 20 mM iodoacetamide, incubated in the dark at 37 °C for 1 h. Then, the sample was precipitated using 300 µl prechilled acetone, incubated at -20℃ overnight. The precipitate was washed twice with cold acetone and resuspended in 50 mM ammonium bicarbonate. Finally, the proteins were digested with sequence-grade modified trypsin (Promega, Madison, WI) at a substrate/enzyme ratio of 50:1 (w/w), incubated at 37 °C for 16 h. Raw Data of DIA was processed and analyzed by Spectronaut Pulsar X (Biognosys AG, Switzerland) with default parameters. The ideal extraction window was determined by using Spectronaut Pulsar X depending on iRT calibration and gradient stability. The average top 3 filtered peptides which passed the 1% Q-value cutoff were used to calculate the major group quantities [24]. After Student’s t-Test, different expressed proteins were filtered if their Q value ≤ 0.05 and absolute AVG log_2_ ratio > 0.58. Proteins were annotated against GO, KEGG and COG/KOG databases [25]. Significant GO functions and pathways were examined within differentially expressed proteins with a Q value ≤ 0.05.

### Metabonomics analysis

Ears were taken at 7 days, 14 days, 21 days and 28 days after pollination. Three biological replicates were used for each stage. Grains were isolated from the center of the ears and frozen immediately. The freeze-dried samples were crushed using a mixer mill (MM 400, Retsch) with a zirconia bead for 1.5 min at 30 Hz. Then 100 mg powder was mixed with 1.0 ml 70% aqueous methanol containing 0.1 mg/L lidocaine for internal standard, incubated overnight at 4 °C. Centrifuge at 10,000 g for 10 min, the supernatant was filtrated (SCAA-104, 0.22-µm pore size; ANPEL, Shanghai, China, www.anpel.com.cn/) before LC–MS/MS analysis. Quality Control (QC) samples were used to detect reproducibility of the experiment. The compounds were analyzed using an LC-ESI-MS/MS system (UPLC, Shim-pack UFLC SHIMADZU CBM30A; MS/MS, Applied Biosystems 6500 QTRAP,). Data filtering, peak detection, alignment, and calculations were performed using Analyst 1.6.1 software.

## Results

### Phenotypic analysis of Chang 7 − 2 and *tc19* grain-related traits

**Fig. 1:**
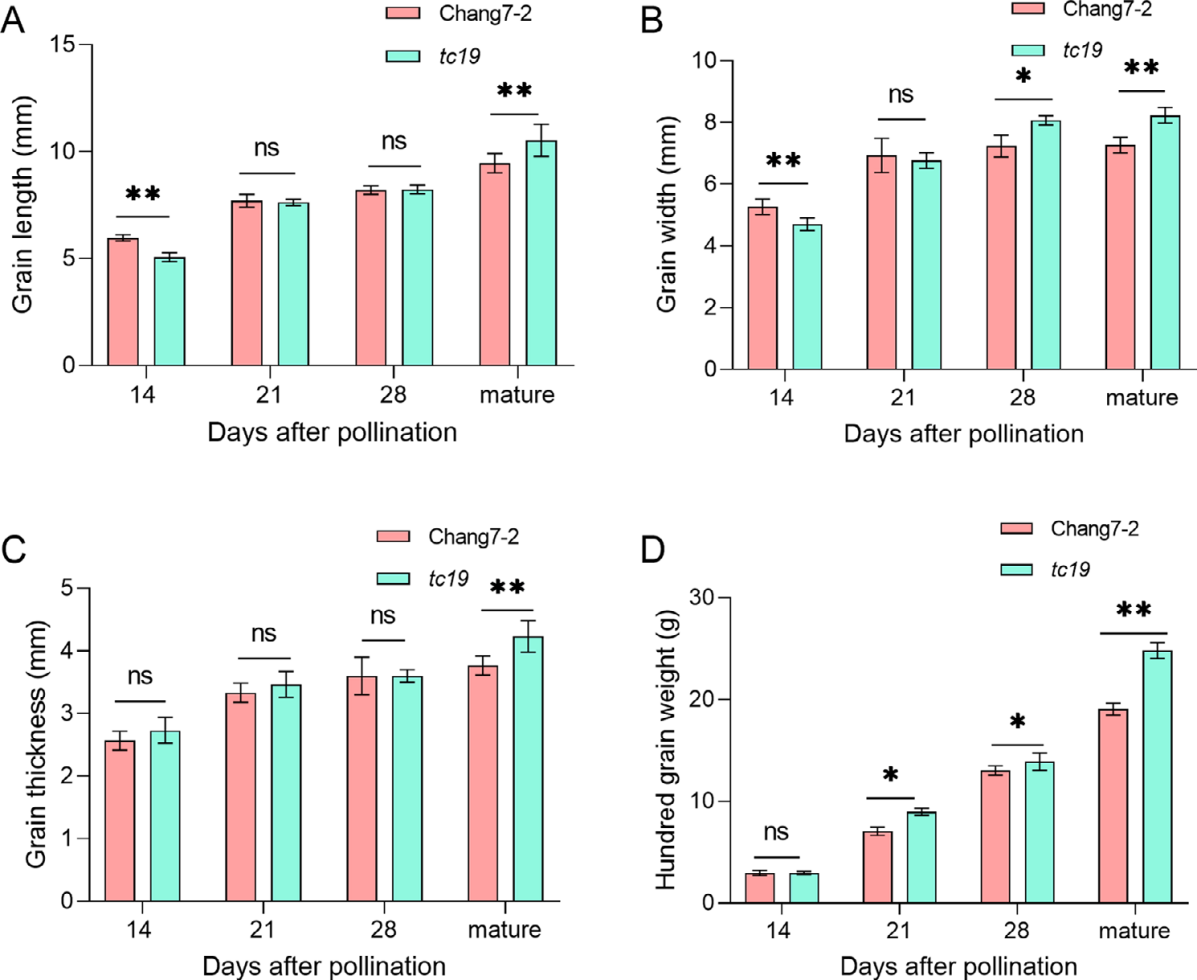
Grain development of Chang7-2 and *tc19*. (**A**) Grain length at different days after pollination. (**B**) Grain width at different days after pollination. (**C**) Grian thickness at different days after pollination. (**D**) Hundred grain weight at different days after pollination. Data are means of three biological replicates. ns, not significant. * p < 0.05. **p < 0.01

We previously identifed a large grain mutant *tc19*, with different grain developmental rates to Chang7-2. First, we repeated the previous phenotype at two years and environments [22]. At 14 DAP, the average grain length of Chang7-2 and *tc19* were respectively 5.81 and 5.31 mm (Fig. 1A). The grain of *tc19* was shorter than that of Chang7-2. After maturity, the average grain length of Chang7-2 and *tc19* were 9.47 and 10.42 mm, respectively. The mature grain of *tc19* is longer than that of Chang7-2. There is no difference between Chang7-2 and *tc19* during 21 and 28 DAP for grain length. This indicates the dynamic change of grain development between Chang7-2 and *tc19*. Additionally, we observed a similar trend in the case of grain width (Fig. 1B). At 14 DAP, the average grain width of Chang7-2 and *tc19* were 5.31 and 4.83 mm, respectively, the grain width of *tc19* was smaller than that of Chang 7-2. After maturity, the grain of *tc19* was wilder than that of Chang7-2. We also analyzed grain thickness (Fig. 1C) and hundred-grain weight (Fig. 1D). After mature, *tc19* was thicker and weighter than Chang7-2. This phenomenon indicates that *tc19* is an ideal material for studing the grain growth rate during the dearly grain developmental stage.

### Proteome characteristics of Chang 7 − 2 and *tc19* during grain development

**Fig. 2:**
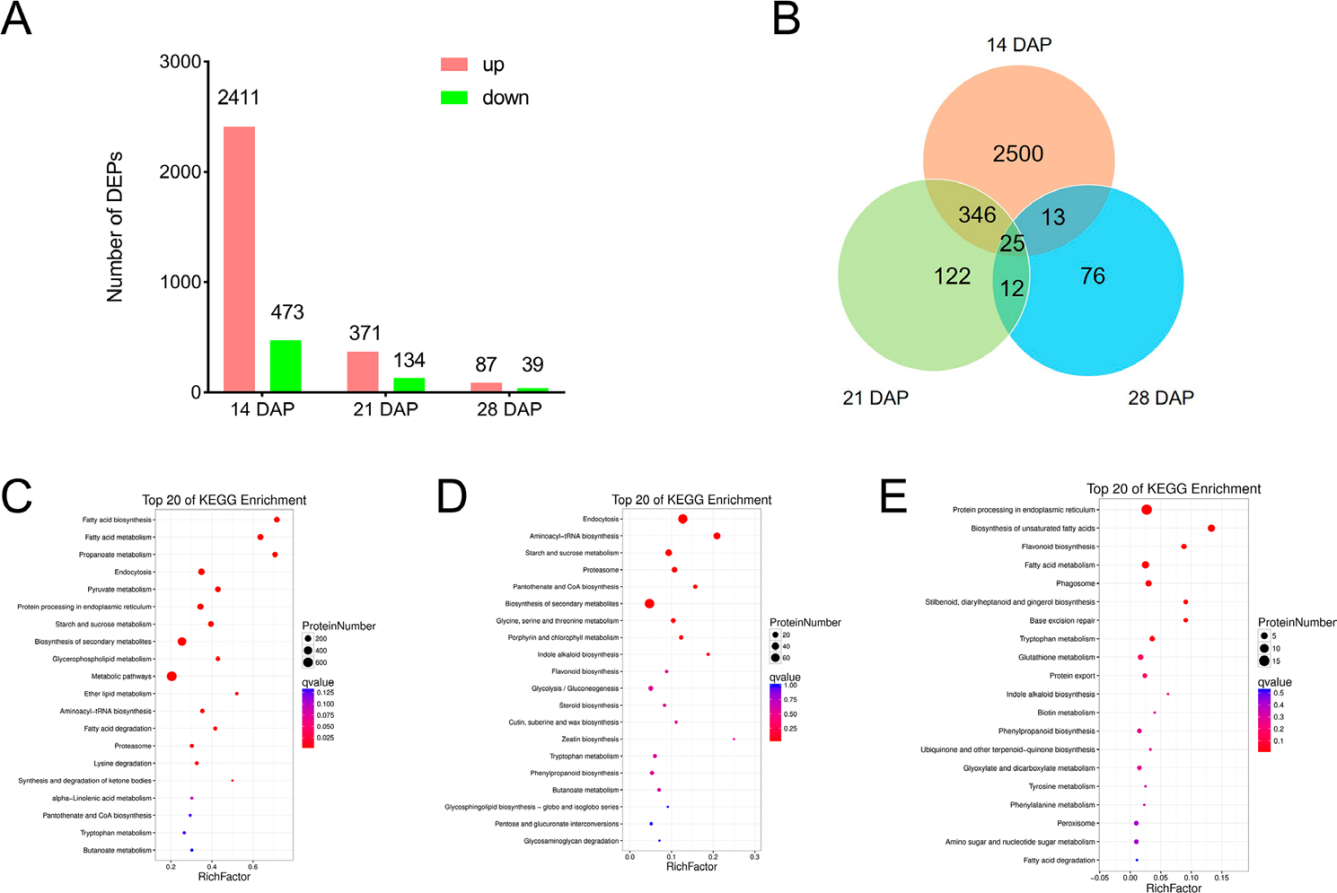
Differentially expressed proteins (DEPs) between Chang7-2 and *tc19*. (**A**) Number of DEPs at different DAPs. DAP, days after pollination. (**B**) Common DEPs at different DAPs. (**C-E**) KEGG analysis of DEPs at 14 DAP. (**D**) KEGG analysis of DEPs at 21 DAP. (**E**) KEGG analysis of DEPs at 28 DAP.

To know which proteins are related to the different grain growth rates between Chang7-2 and *tc19*, we performed the proteomic analysis at 14, 21 and 28 DAP. At 14 DAP, we identified 2884 DEPs between Chang7-2 and *tc19*, of which 2411 were up-regulated while 473 were down-regulated in *tc19*. At 21 DAP, 505 DEPs were identified, 371 were up-regulated and 134 down-regulated in *tc19*. At 28 DAP, 126 DEPs were identified, of which 87 were up-regulated and 39 were down-regulated in *tc19* (Fig. 2A). The results indicated that all three stages were affected for grain development in *tc19*. DEPs common for at least two stages were identified (Fig. 2B). 371 DEPs were found in the comparasions at both 14 DAP and 21 DAP, 37 DEPs were found in the comparasions at both 21 DAP and 28 DAP, and 25 DEPs were found at all the three seeds developmental stages mentioned above.

GO enrichment analysis showed that the terms of metabolic process, ceullar process and binding were the most significantly enriched at 14 DAP, while the terms of metabolic process, marcromolecular complex and binding were the most significantly enriched at 21 DAP, the terms of metabolic process, binidng and catalytic activity were the most significantly enriched at 28 DAP (Supplementary Fig. S1). KEGG analysis showed that the pathways of metabolic pathway, biosynthesis of secondary metabolites and endocytosis were among those that were significantly enriched at 14 DAP (Fig. 2C). The pathways of biosynthesis of secondary metabolites and endocytosis were among those that were significantly enriched at 21 DAP (Fig. 2D). The pathways of protein processing in endoplasmic reticulum, biosynthesis of unsaturated fatty acids and fatty acid metabolism were among those that were significantly enriched at 28 DAP (Fig. 2E). In summary, DEPs were most involved in metabolic processes.

### Metabolome analysis during the grain development of Chang 7 − 2 and *tc19*

**Fig. 3:**
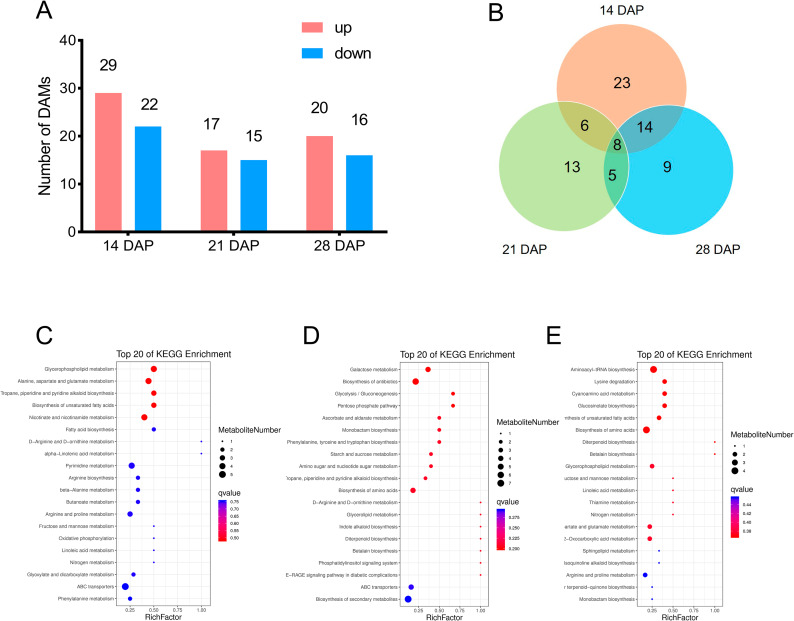
Differentially accumulates metabolites (DAMs) between Chang7-2 and *tc19* at different DAPs. DAP, days after pollination. (**A**) Number of DAMs at different DAPs. (**B**) Common DAMs at different DAPs. (**C-E**) KEGG analysis of DAMs at 14 DAP. (**D**) KEGG analysis of DAMs at 21 DAP. (**E**) KEGG analysis of DAMs at 28 DAP.

To know how the accumulation of metabolites was affected in *tc19*, we performed a metabolic analysis following the method described previously [26]. For all three stages, Chang7-2 and *tc19* had 78 differentially accumulated metabolites (DAMs) which could be classified into 10 categories, including lipids, phenolic acids, alkaloids, nucleotides and their derivatives, amino acids and their derivatives compounds, organic acids, terpenes, lignin and coumarin, flavonoids and others. At 14 DAP, 51 DAMs were identified, with 29 up-regulated and 22 down-regulated in *tc19*. At 21DAP, 32 DAMs were identified, with 17 up-regulated and 15 down-regulated in *tc19*. At 28 DAP, 36 DAMs were identified, with 20 up-regulated and 16 down-regulated in *tc19* (Fig. 3A). Common DAMs were identified (Fig. 3B). 14 DAMs were identified at both 14 DAP and 21 DAP, 13 DAMs were identified at both 21 DAP and 28 DAP. 8 DAMs were identified at all the three stages, including pipecolic acid, trigonelline, N-acetylputrescine, 6-deoxyfagomine, N-benzylmethylene isomethylamine, protocatechuic acid-4-glucoside, 3-O-(E)-p-coumaroyl quinic acid, trihydroxycinnamoylquinic acid. 5 DAMs were identified at 21 DAP and 28 DAP, but not at 14 DAP, including L-(-)-Tyrosine, Phenylalanine, 3-Hydroxypropyl palmitate glc-glucosamine, 1-O-β-D-Glucopyranosyl sinapate and Ferruginol. KEGG analysis showed that the DAMs are involved in different pathways (Fig. 3C, D and E).

### Correlation analysis of Transcriptomics and Proteomics of Chang 7 − 2 and *tc19* grain

**Fig. 4:**
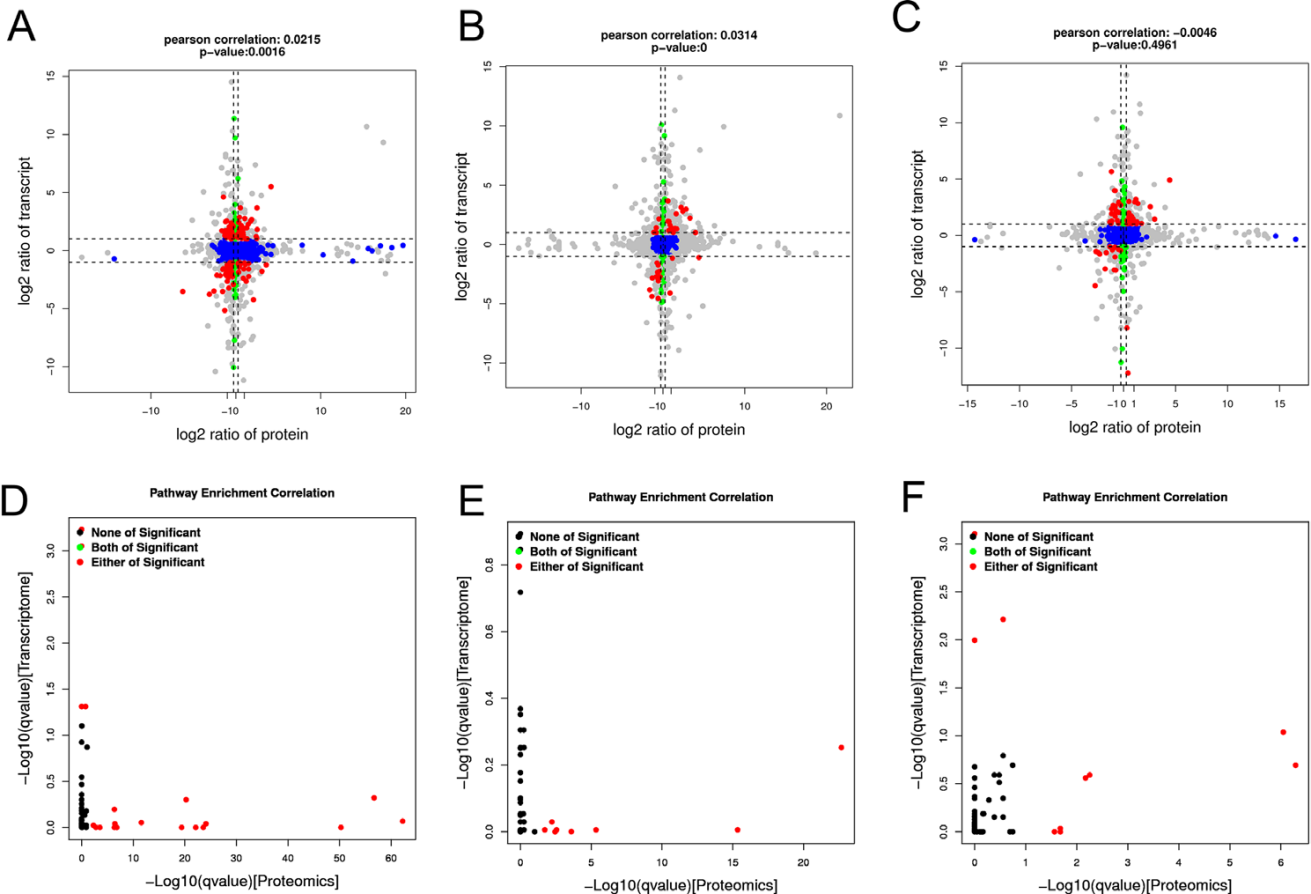
Comparative analysis between DEGs and DEPs in Chang7-2 and *tc19*. (**A**) Pearson correlation of DEGs and DEPs at 14 days after pollination. (**B**) Pathway enrichment correlation between DEGs and DEPs at 14 days after pollination. (**D**) Pearson correlation of DEGs and DEPS at 21 days after pollination. (**E**) Pathway enrichment correlation between DEGs and DEPs at 21 days after pollination. (**F**) Pearson correlation of DEGs and DEPS at 28 days after pollination. (**G**) Pathway enrichment correlation between DEGs and DEPs at 28 days after pollination

To know how DEGs and DEPs are associated with grain development in *tc19*, we performed DEGs and DEPs correlation analysis and generated nine-quadrant plots. At 14 DAP, we identified 2,987 and 4,484 DEGs and DEPs, respectively. There were 130 genes showing consistent transcription and translation trends, 53 up-regulated and 77 down-regulated in *tc19*. We identified 118 genes showing opposite transciption and translation trends, 54 up-regulated and 64 down-regulated in *tc19* (Fig. 4A). KEGG analysis found that 60 genes are enriched in 32 different pathways (Fig. 4B).

At 21 DAP, we identified 2,647 and 1,083 DEGs and DEPs, respectively. We found 78 genes showing consistent transcriptional and translational trends, 48 upregulated and 30 down regulated in *tc19*. We found 26 genes showed opposite transcription and translation trends, 23 upregulated and 3 downregulated in *tc19* (Fig. 4C). KEGG analysis found that 37 interelated genes were enriched in 18 pathways (Fig. 4D).

At 28 DAP, we identified 3,209 and 216 DEGs and DEPs, respectivley. Among them, 40 had the same transcription and translation trend, 25 upregulated and 15 downregulated in *tc19*. We identified 7 genes showing opposite translation and translation trends, 4 upregulated and 3 downrelated in *tc19* (Fig. 4E). KEGG analysis found 16 interrelated genes enriched in 11 pathways (Fig. 4F).

### Comparative analysis of transcriptomics, proteomics and metabolomics of Chang 7 − 2 and *tc19*

**Fig. 5:**
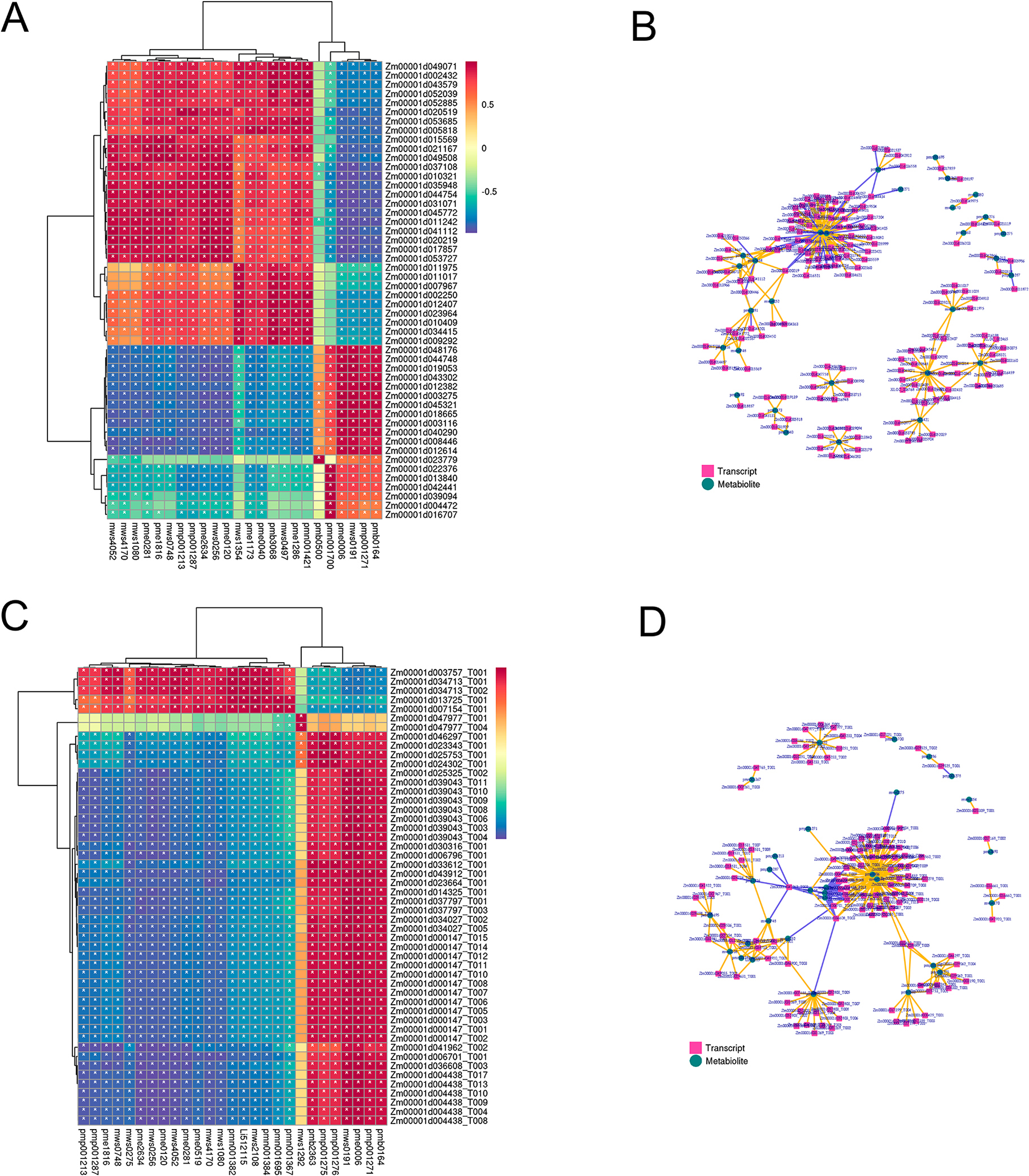
Comparative analysis between DEGs and DAMs, DEPs and DAMs, respectively, in Chang7-2 and *tc19*. (**A**) A heat map of pearson correlation of DEGs and DAMs. (**B**) A diagram of correlated DEGs and DAMs. (**C**) A heat map of pearson correlation of DEGs and DAMs. (**D**) A diagram of correlated DEGs and DAMs.

To know which and how the DEGs affected the accumulation of DAMs, we calculated the Pearson correlation coefficient between DEGs and DAMs. The correlation between the top 50 DEGs and DAMs with correlation coefficients is shown in heat maps (Fig. 5A). The correlation between gene expression and metabolite abundance is shown in the network diagram (Fig. 5B). Additionally, to know how the DEPs affected the accumulation of DAMs, we calculated Pearson correlation coefficient between DEPs and DAMs. The correlation between the top 50 DEGs and DAMs with correlation coefficients is shown in heat maps (Fig. 5C). The correlation between gene expression and metabolite abundance is shown in the network diagram (Fig. 5D). We found a couple of DAMs significantly correlated to both DEGs and DEPs, such as mws4170, mws1080, pme1816, pme2634, mws0748.

### The starch biosynthesis and the phenylpropanoid pathway are affected in *tc19*

To test if the starch biosynthesis was changed in *tc19*, we analyzed the DEGs, DEPs and DAMs in starch biosynthesis. We found UDP-glucose upregulated while D-Glucose downregulated in *tc19*. In rice, UDP-glucosyltransferase regulates grain size by modulating cell proliferation and expansion, which are regulated by flavonoid-mediated auxin levels and related gene expression [27]. Our data is consistent with the study. Specifically, we found glycogen phosphorylase (Zm00001d034074), 1,4-alpha-glucan branching enzyme (Zm00001d003817), UTP-glucose-1-phosphate uridylyltransferase (Zm00001d015008), beta-glucosidase (Zm00001d028199, Zm00001d022367), beta-fructofuranosidase (Zm00001d016708) were affected at the proteomic level in *tc19*.

**Fig. 6:**
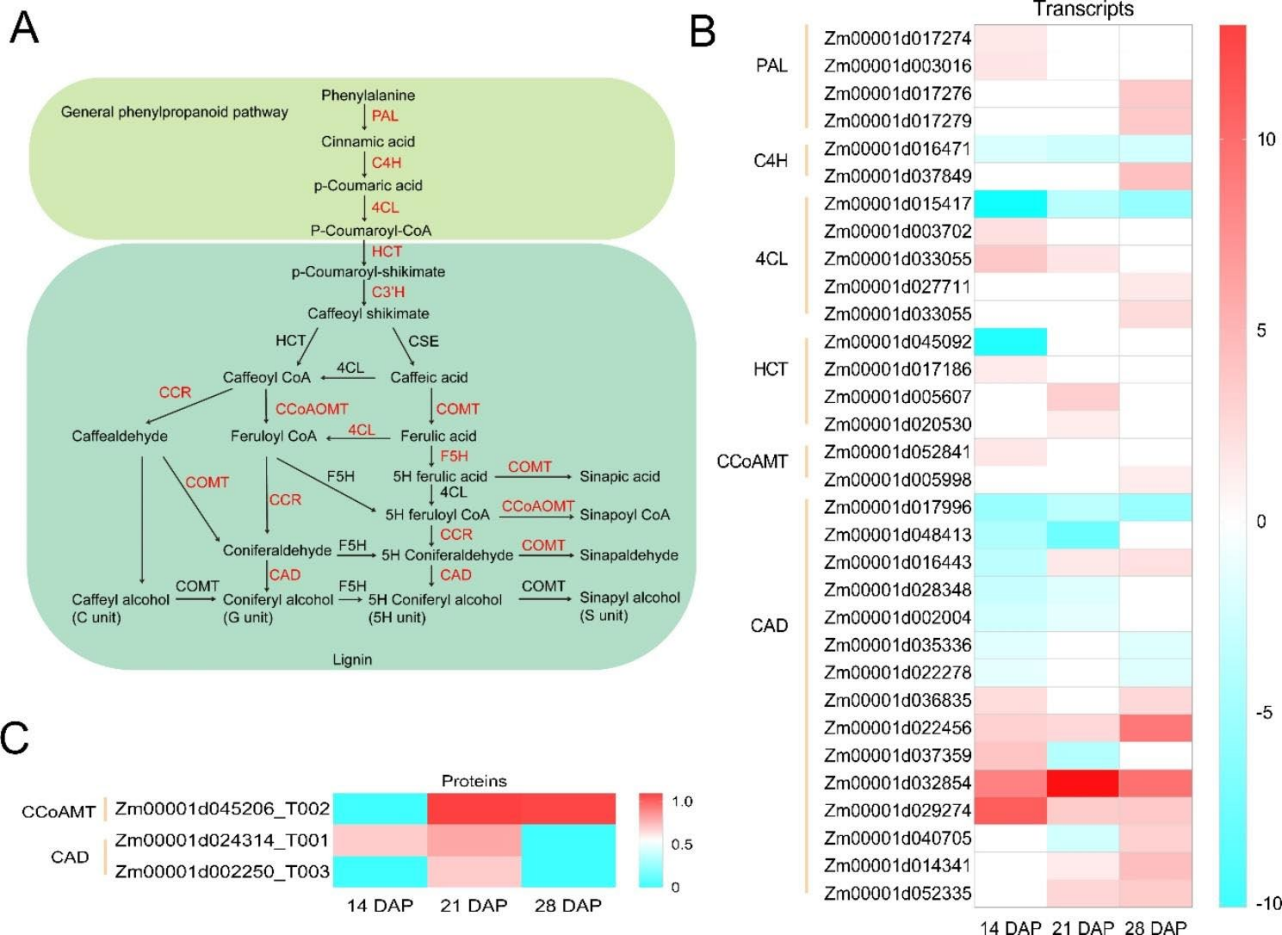
Differentially expressed genes (DEGs), differentially expressed proteins (DEPs) and differentially accumulated metabolites (DAMs) involved in phenylpropanoid pathway between Chang7-2 and *tc19*. (**A**) Simplified model of phenylpropanoid pathway (Dong et al., 2021). Red color means DEGs or DEPs between Chang7-2 and *tc19*. (**B**) Heatmap of DEGs between Chang7-2 and *tc19*. (**C**) Heatmap of DEPs between Chang7-2 and *tc19*. The color scale indicates the log2(fold-change) of the FPKM transcripts, proteins and metabolites values in Chang7-2 relative to *tc19*. Gene ID refers to Zm00001d identifies

We found many DAMs, DEGs and DEPs invovled in the phenylpropanoid pathway (Dong et al., 2021). Some metabolites, such as tyrosine, ferulic acid and sinapate, were upregulated in *tc19* (Fig. 6A). Many genes changed at the transcriptomic level, for example, PAL (phenylalanine ammonia lyase), C4H (Cinnamate 4-Hydroxylase), 4CL (4-coumarate: coenzyme A ligase), HCT (hydroxycinnamoyl-Coenzyme A shikimate/quinate hydroxycinnamoyltransferase), CCoAMT (caffeoyl-CoA 3-O-methyltransferase) and CAD (cinnamyl-alcohol dehydrogenase) (Fig. 6B). CCoAMT and CAD also changed at the proteomic level (Fig. 6C). This indicates that the phenylpropanoid pathway is dramatically affected in *tc19*, which may explain the large grain phenotype of *tc19*. However, we didn’t measure phenylpropanoid biosynthesis related physiologic tratis in *tc19*. More experiments need to be carried out in the future.

## Disscussion

Previous studies have revealed the importance of flavonoids and ligin in grain size. Flavonoids and lignin are biosynthesized from the phenyplropane pathway [28]. The biosynthesis of phenylpropane starts with phenylalanine and tyrosine. We found phenylalanine is downregulated while tyrosine is upregualted at 21 and 28 DAP (Fig. 3). The grain growth rates of *tc19* are faster than those of Chang7-2 at 21 and 28 DAP (Fig. 1). Considering that phenylalanine and tyrosine were not significantly affected at 14 DAP (Fig. 3), which is consistent with the slower grain growth rates in *tc19* at 14 DAP (Fig. 1). Recently, several studies revealed that phenylpropane compounds play important roles in grain size [29–31]. This indicates that phenylalanine and tyrosine play important roles in *tc19* grain growth rate.

Starch and protein accumulation is believed to be the main factors affecting grain size and weight [3]. We found that tyrosine biosynthesis and degradation, leucine and flavanone biosynthesis were consistent between transcriptomic, proteomic and metabolic data. We found several DEGs (Fig. 3). Among them, *TYRAAT2* encodes arogenate dehydrogenase involved in tyrosine biosynthesis [32]. *GSTZ1* encodes glutathione S-transferase Z1 which is involved in tyrosine degradation [33]. *IPMS2* encodes 2-isopropylmalate synthase 1, which is involved in leucine biosynthesis [34]. *4CLL* encodes 4-coumarate-CoA ligase like 7, involved in flavanone biosynthesis [35]. However, it is not clear how these genes influence grain development. The biosynthesis priority between protein and starch may have important roles in grain development [27].

Carbon metabolism provides the necessary energy for various metabolic pathways [36]. We identified several DEPs involved in carbohydrate metabolisms, such as endo-β-1,3-glucanases and 1,3,4-inositol triphosphate 5/6-kinases (Fig. 3). At 14 DAP, the expression level of the endo-β-1,3-glucanases in Chang7-2 was higher than that in *tc19*, while at 28 DAP, the expression level of Chang7-2 was lower than that in *tc19*. In contrast, the expression of 1,3,4-inositol triphosphate 5/6-kinases in Chang7-2 was lower than that of *tc19* at 14 DAP, while higher at 28 DAP in Chang7-2 than that in *tc19*. These indicate that the expression of endo-β-1,3-glucanases and 1,3,4-inositol triphosphate 5/6-kinases are related to the grain growth rate of *tc19*.

## Conclusion

Using the Chinese elite maize breeding line Chang7-2 and its large grain mutant *tc19*, we performed the comparative transcriptomic, proteomic and metabolomic analysis at different grain development stages. Through proteomics analyses, we found 2884, 505 and 126 differentially expressed proteins (DEPs) at 14, 21 and 28 days after pollination, respectively. Through metabolomics analysis, we found 51, 32 and 36 differentially accmulated metabolites (DAMs) at 14, 21 and 28 days after pollination, respectively. Through multiomics comparative analysis, we found the phenylpropanoid pathways are influenced at transcriptomic, proteomic and metabolomic levels in all the three grain developmental stages. We identified several genes in phenylpropanoid biosynthesis, which may explain the large grain phenotype of *tc19*. To confirm the imporantance of phenylpropanoid biosynthesis in grain development, it will be necessary to measure phenylpropanoid biosynthesis related physiologic tratis in the future.

### Electronic supplementary material

Below is the link to the electronic supplementary material.

Supplementary Material 1

Supplementary Material 2

Supplementary Material 3

## Data Availability

The raw RNA sequence data are available in the NCBI Sequence Read Archive (SRA) repository. The accession number is PRJNA724904, the website link is https://dataview.ncbi.nlm.nih.gov/object/PRJNA724904. The raw protein sequence data are available in the iProX data license, the accession number is IPX0006305000, the website link is https://www.iprox.cn/page/SSV024.html;url=1688606344391eMsl. All data supporting the conclusions of this article are included in the article and its additional fles.
