# Supplementary figures and images for: Multiomics comparative analysis of the maize large grain mutant tc19 identified pathways related to kernel development
Source: BMC Genomics. 2023 Sep 11;24:537. doi: 10.1186/s12864-023-09567-z (PMC10496403; doi:10.1186/s12864-023-09567-z)

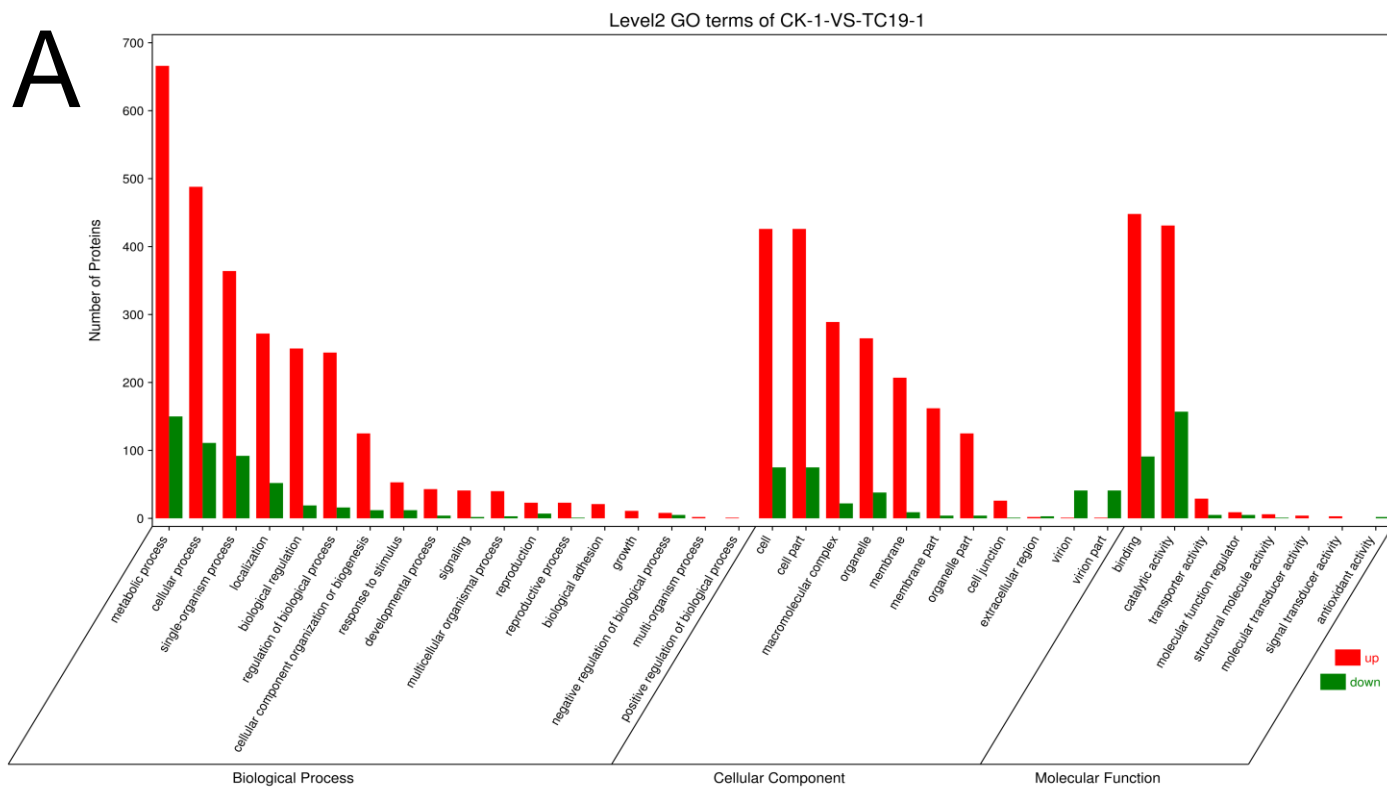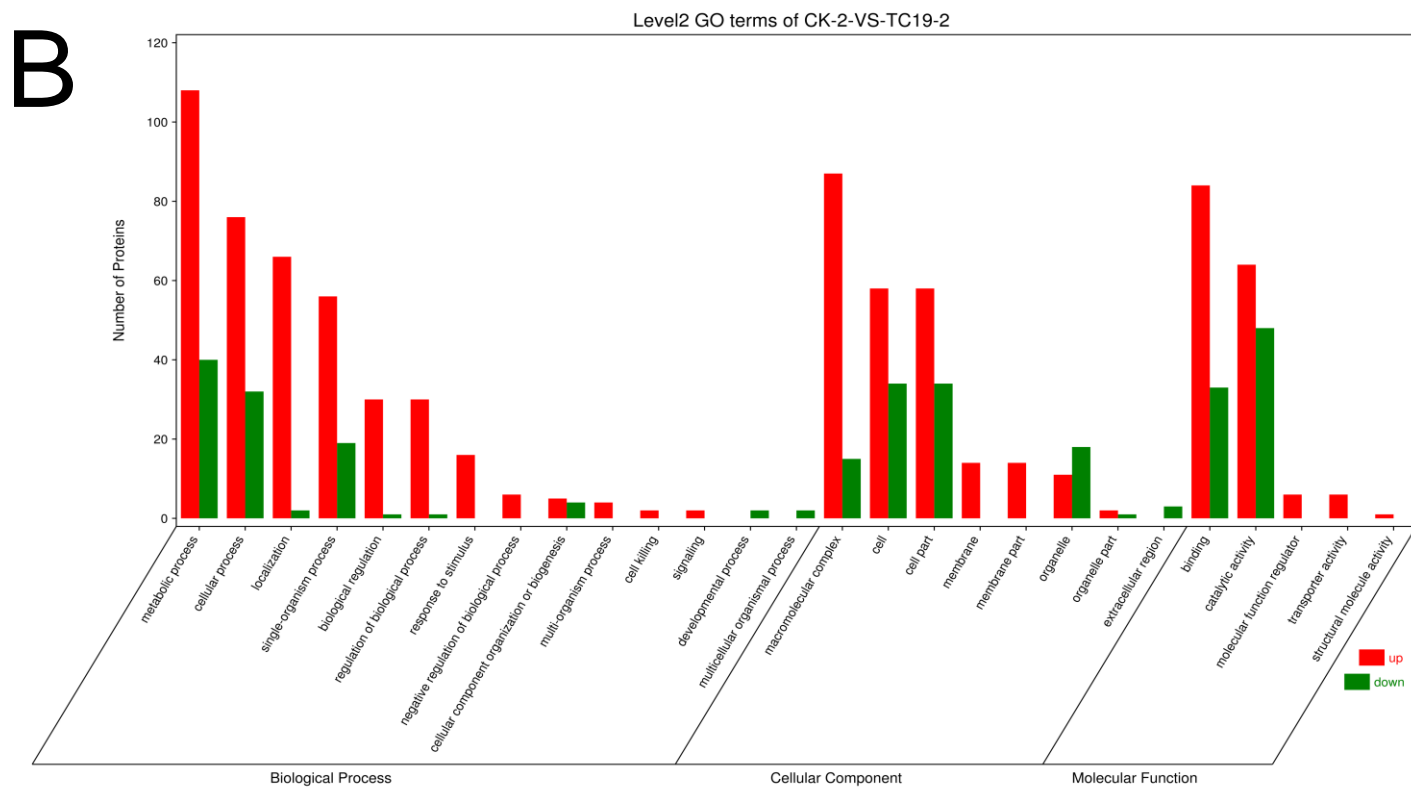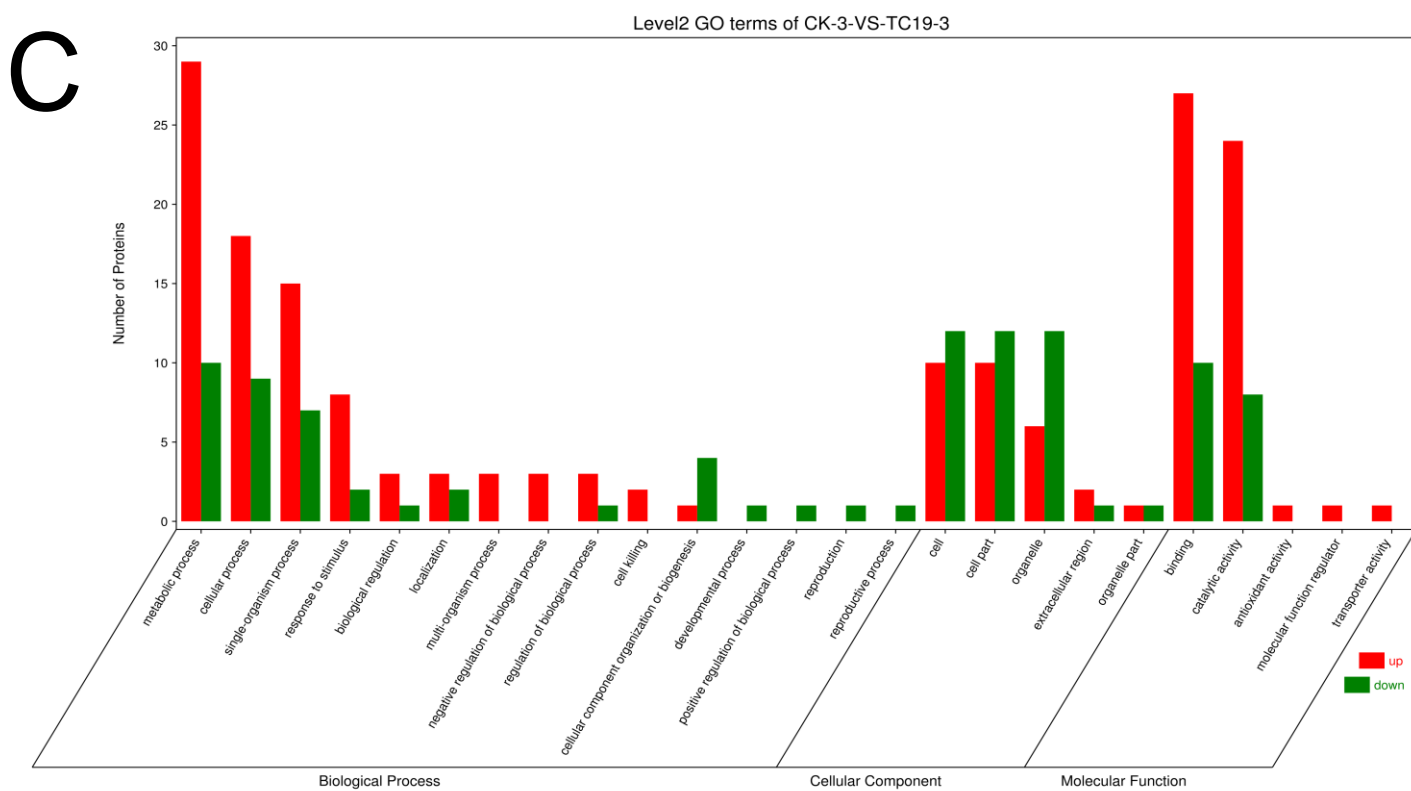

Fig. S1 Enriched Go terms for DEPs in Chang7-2 and *tc19*

Supplement: Supplementary file 1 — Supplementary Material 1 [file 12864_2023_9567_MOESM1_ESM.pdf]
